# Targeting Iron Metabolism and Ferroptosis as Novel Therapeutic Approaches in Cardiovascular Diseases

**DOI:** 10.3390/nu15030591

**Published:** 2023-01-23

**Authors:** Yufei Chen, Xueting Li, Siyuan Wang, Ran Miao, Jiuchang Zhong

**Affiliations:** 1 Heart Center and Beijing Key Laboratory of Hypertension, Beijing Chaoyang Hospital, Capital Medical University, Beijing 100020, China; 2 Department of Cardiology, Beijing Chaoyang Hospital, Capital Medical University, Beijing 100020, China; 3 Medical Research Center, Beijing Institute of Respiratory Medicine and Beijing Chaoyang Hospital, Capital Medical University, Beijing 100020, China

**Keywords:** iron metabolism, ferroptosis, lipid peroxidation, cardiovascular diseases, iron chelator

## Abstract

Iron functions as an essential micronutrient and participates in normal physiological and biochemical processes in the cardiovascular system. Ferroptosis is a novel type of iron-dependent cell death driven by iron accumulation and lipid peroxidation, characterized by depletion of glutathione and suppression of glutathione peroxidase 4 (GPX4). Dysregulation of iron metabolism and ferroptosis have been implicated in the occurrence and development of cardiovascular diseases (CVDs), including hypertension, atherosclerosis, pulmonary hypertension, myocardial ischemia/reperfusion injury, cardiomyopathy, and heart failure. Iron chelators deferoxamine and dexrazoxane, and lipophilic antioxidants ferrostatin-1 and liproxstatin-1 have been revealed to abolish ferroptosis and suppress lipid peroxidation in atherosclerosis, cardiomyopathy, hypertension, and other CVDs. Notably, inhibition of ferroptosis by ferrostatin-1 has been demonstrated to alleviate cardiac impairments, fibrosis and pathological remodeling during hypertension by potentiating GPX4 signaling. Administration of deferoxamine improved myocardial ischemia/reperfusion injury by inhibiting lipid peroxidation. Several novel small molecules may be effective in the treatment of ferroptosis-mediated CVDs. In this article, we summarize the regulatory roles and underlying mechanisms of iron metabolism dysregulation and ferroptosis in the occurrence and development of CVDs. Targeting iron metabolism and ferroptosis are potential therapeutic strategies in the prevention and treatment of hypertension and other CVDs.

## 1. Introduction

Iron (Fe) is an essential micronutrient in the human body and fulfills a variety of functions in biochemical and physiological processes, including the transportation and storage of oxygen, mitochondrial respiration, and redox reactions [1,2]. The human body contains about 2–5 g of total iron, most of which is bound intracellularly to heme in hemoglobin and myoglobin or to other non-heme proteins and enzymes [3,4]. Extracellular iron contains approximately only 0.1% of total body iron, most of which is bound to the iron transport protein transferrin (Tf) in serum [3,4]. Disturbances in iron homeostasis, including iron overload and iron deficiency, cause various diseases such as iron deficiency anemia and thalassemia. Ferroptosis, an iron-dependent newly identified regulated cell death induced by iron overload and lipid peroxidation, has been implicated in the pathogenesis of cardiovascular disorders [5,6,7].

**Figure 1:**
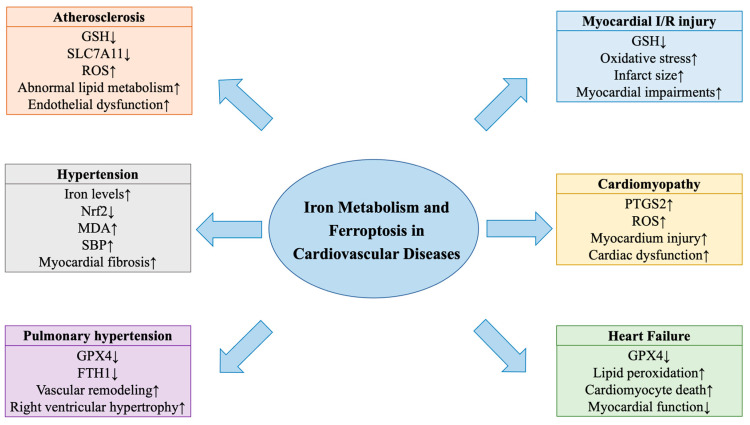
Iron metabolism and ferroptosis in cardiovascular diseases. FTH1, ferritin heavy chain 1; GPX4, glutathione peroxidase 4; GSH, glutathione; I/R, ischemia/reperfusion; MDA, malondialdehyde; Nrf2, nuclear factor erythroid 2-related factor 2; PTGS2, prostaglandin endoperoxide synthase 2; ROS, reactive oxygen species; SBP, systolic blood pressure; SLC7A11, subunit solute carrier family 7 member 11; ↑upregulation, ↓downregulation.

Currently, cardiovascular diseases (CVDs) are the leading causes of mortality and morbidity worldwide in noncommunicable diseases [8]. Recently, accumulating evidence confirmed the crucial roles of iron metabolism and ferroptosis in the physiology and pathophysiology of cardiovascular dysfunctions, including atherosclerosis [9,10,11], hypertension [12], pulmonary hypertension (PH) [13], myocardial ischemia/reperfusion (I/R) injury [14,15], cardiomyopathy [16,17], and heart failure [18] (Figure 1). In this article, we focus on the impacts and underlying mechanisms of iron metabolism and ferroptosis in the pathogenesis of CVDs and highlight the potential therapeutic value of targeting iron metabolism and ferroptosis in CVDs.

## 2. Iron Metabolism and Ferroptosis

**Figure 2:**
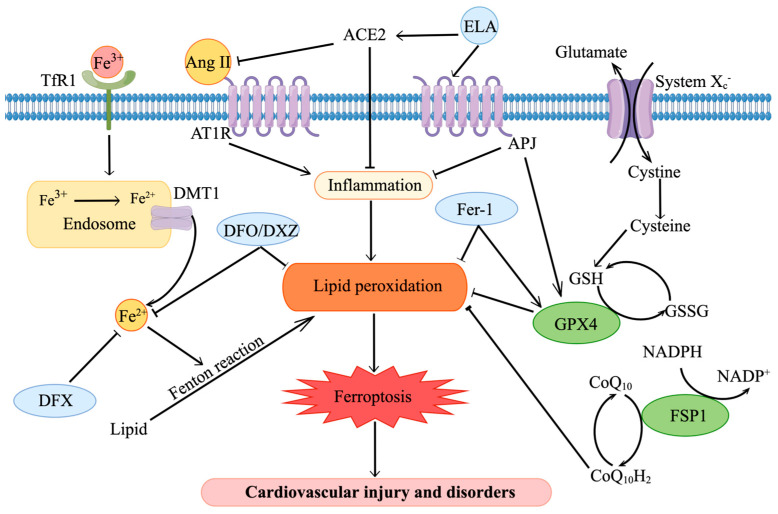
The interplay between iron metabolism and ferroptosis in cardiovascular system. ACE2, angiotensin-converting enzyme 2; Ang II, angiotensin II; APJ, apelin receptor; AT1R, Ang II type 1 receptor; CoQ_10_, coenzyme Q_10_; DFO, deferoxamine; DFX, deferasirox; DXZ, dexrazoxane; DMT1, divalent metal transporter 1; ELA, elabela; Fe^2+^, ferrous iron; Fe^3+^, ferric iron; Fer-1, ferrostatin-1; FSP1, ferroptosis suppressor protein 1; GPX4, glutathione peroxidase 4; GSH, glutathione; GSSG, glutathione disulfide; NADPH, nicotinamide adenine dinucleotide phosphate; TfR1, transferrin receptor 1. This figure was created using Figdraw (www.figdraw.com), accessed on 6 November 2022.

Iron homeostasis is essential for the normal physiological activity of cells and systems. Redundant intracellular iron, especially Fe^2+^, induces lipid peroxidation via the Fenton reaction, resulting in ferroptosis (Figure 2) [19,20]. Generally, the absorption, utilization and output of iron regulate the balance of iron homeostasis. Dietary iron is absorbed into the body in form of heme iron or non-heme iron [21]. Compared to non-heme iron, heme iron is more readily absorbed. No-heme inorganic ferric iron (Fe^3+^) is firstly reduced to Fe^2+^ by enzymes and then transported into cells through divalent metal transporter 1 (DMT1) at the apical membrane of the enterocyte [22,23]. Enterocyte Fe^2+^ is exported to plasma through ferroportin (FPN) at the basal membrane [22]. Fe^2+^ is then oxidized to Fe^3+^ and binds with Tf to form a Tf-Fe^3+^ complex [24]. The circulating Tf-Fe^3+^ complex is carried to different cells and is endocytosed into cells through transferrin receptor 1 (TfR1) (Figure 2) [4,22,24]. Mice lacking TfR1 in the heart had severe cardiomegaly and poor myocardial function, which could be blocked by aggressive iron therapy, suggesting the role of TfR1 in maintaining intracellular iron levels and the significance of iron hemostasis in the myocardium [25]. Imported Fe^3+^ is transported to the endosome and reduced to Fe^2+^ by the six-transmembrane epithelial antigen of prostate 3 (STEAP3), and is then transported into the cytoplasm via DMT1 (Figure 2) [2,19,26]. FPN is the only protein responsible for the efflux of excess iron in cells and can be degraded by hepcidin [27,28]. Mice with cardiomyocyte-targeted deletion of FPN had myocardium dysfunction with iron accumulated in cardiomyocytes [29]. Knockdown of FPN promoted iron accumulation and oxidative reaction in a lipopolysaccharide (LPS)-induced endotoxemia rat model, and was implicated in ferroptosis and new-onset atrial fibrillation [30]. Hepcidin inhibited macrophage-mediated myocardium repair and regeneration after cardiac impairments [31]. The hepcidin-FPN axis plays a key role in the maintenance of iron homeostasis. Iron content and inflammation influence the production of hepcidin [32]. Iron overload and chronic inflammation were suggested to be associated with elevated levels of hepcidin, which blunted the release of iron and promoted iron being trapped in cells, thereby leading to intracellular iron restriction [32]. In contrast, hepcidin was reduced in states of iron deficiency or hypoxia, resulting in more iron being released into the circulation [32]. 

Intracellular ferritin regulates iron homeostasis by binding iron. The binding of iron to ferritin inhibits iron-mediated oxidative activation and ferroptosis [4]. Poly(rC)-binding protein 1 (PCBP1) is a cytosolic iron chaperone that carries Fe^2+^ to ferritin [20]. Deletion of PCBP1 in mouse hepatocytes upregulated labile iron and accumulated reactive oxygen species (ROS), leading to ferroptosis [33]. PCBP1 knockdown in head and neck cancer cells promoted ferritinophagy-mediated ferroptosis [34]. Iron-bound ferritin can be degraded by nuclear receptor coactivator 4 (NCOA4)-mediated autophagic degradation named ferritinophagy, accompanied by the release of Fe^2+^ [24]. Deletion of NCOA4 in mouse hearts mitigated cardiac dysfunction and ferritinophagy-mediated ferritin degradation induced by pressure overload [20,35]. Cytoplasmic proteins including iron regulatory protein 1 (IRP1) and IRP2 regulate intracellular iron levels at posttranscriptional levels [20]. The binding of IRP1 and IRP2 to the 3′-untranslated region (UTR) of TFR1 mRNA promotes their translation, while the binding of IRP1 and IRP2 to 5′-UTR of FPN or ferritin heavy chain 1 (FTH1) inhibits their translation [20]. Cardiomyocyte-specific deletion of IRP1 and IRP2 in mice was associated with more severe myocardial dysfunction and increased the mortality of heart failure after myocardial infarction, accompanied by impaired mitochondrial respiration [35]. Nuclear factor erythroid 2-related factor 2 (Nrf2) acts as a regulator of antioxidant responses, playing a critical role in the transcriptional regulation of several iron metabolism-associated genes including FTH, Tf, FPN, and heme oxygenase-1 (HMOX1) [4,36]. HMOX1 was involved in ferroptosis-mediated doxorubicin (DOX)-induced cardiomyopathy (DIC) through the accumulation of non-heme iron in response to Nrf2, which could be rescued by a HMOX1 antagonist or Nrf2-deficiency in mice [37].

Ferroptosis, a unique form of iron-dependent nonapoptotic cell death induced by the small molecule erastin, was firstly identified by Dixon in 2012 [7]. In morphology, ferroptotic cells are characterized by morphological changes such as mitochondrial shrinkage, increased mitochondrial membrane density, decreased or absent mitochondrial cristae and loss of mitochondrial outer membrane integrity [24,38]. Iron accumulation is associated with the overproduction of ROS and contributes to ferroptosis. Moreover, intracellular depletion of glutathione (GSH) and the inactivation of glutathione peroxidase 4 (GPX4) lead to the accumulation of membrane lipid peroxidation, resulting in ferroptosis [24,38]. The System X_c_^–^–GSH–GPX4 pathway plays a major role in the regulation of oxidative stress-mediated ferroptosis (Figure 2) [3]. System X_c_^–^ is composed of subunit solute carrier family 7 member 11 (SLC7A11) and solute carrier family 3 member 2 (SLC3A2), which contributes to the synthesis of GSH through the exchange of cystine and glutamate across the plasma membrane (Figure 2) [39]. GSH is an efficient intracellular antioxidant synthesized from cysteine, glutamate and glycine [24]. After reacting with lipid peroxides, GSH is oxidized to glutathione disulfide (GSSG) [28]. GSH-dependent GPX4 converts toxic lipid hydroperoxides (L-OOH) to non-toxic lipid alcohols (L-OH) within cell membranes and blocks lipid peroxidation (Figure 2) [38,40]. GPX4 plays a crucial role in the antioxidant system and the suppression of ferroptosis in CVDs. Overexpression of GPX4 in ApoE^−/−^ mice inhibited lipid peroxidation and ferroptosis (Table 1) [41]. In contrast, the heterodeletion of GPX4 aggravated ferroptosis and cardiac impairments [42]. The ferroptosis suppressor protein 1 (FSP1)–coenzyme Q_10_ (CoQ_10_)–nicotinamide adenine dinucleotide phosphate (NADPH) pathway is another antioxidant mechanism which suppresses phospholipid peroxidation and ferroptosis in an independent parallel manner (Figure 2) [3,39,43]. FSP1 functions as an oxidoreductase that reduces CoQ_10_ to ubiquinol via NADPH and inhibits ferroptosis (Figure 2) [39]. 

## 3. Iron Metabolism, Ferroptosis and Cardiovascular Diseases

CVDs account for the main causes of morbidity and mortality in the world. Emerging evidence has evidenced the crucial role of iron metabolism and ferroptosis in the occurrence and development of CVDs. The interplay between iron metabolism and ferroptosis has been implicated in the pathogenesis of vascular diseases and heart diseases.

### 3.1. Iron Metabolism and Ferroptosis in Atherosclerosis

Atherosclerosis is a chronic inflammatory vascular disease characterized by abnormal lipid metabolism and endothelial dysfunction [11,47]. Iron metabolism plays a key role in the development of atherosclerosis. Iron overload contributes to ROS overproduction and is associated with inflammatory responses and lipoprotein changes, which are significant in the pathogenesis of atherosclerosis [48]. Macrophage polarization is regulated by intracellular iron levels, and different subtypes of macrophages determine the size and stability of plaques in atherosclerosis [39,49]. Interleukin-1β (IL-1β) is crucial to the pathogenesis of atherothrombotic plaques [10]. Interestingly, the IL-1β/interleukin-6 (IL-6) signaling pathway regulates iron metabolism by modulating hepcidin levels [50]. 

Ferroptosis has been implicated in the development of atherosclerosis. Overexpression of GPX4 in ApoE^−/−^ mice inhibited the progression of atherosclerosis by suppressing lipid peroxidation (Table 1) [41]. In high-fat diet-induced ApoE^−/−^ mice, ferrostatin-1 (Fer-1) mitigated atherosclerosis by preventing ferroptosis and reducing iron accumulation and lipid peroxidation, suggesting the role of ferroptosis in atherosclerosis (Figure 1, Table 1) [9]. In oxidized low-density lipoprotein-treated human coronary artery endothelial cells, the overexpression of prenyldiphosphate synthase subunit 2 (PDSS2) blunted ferroptosis by suppressing the accumulation of iron and reducing the production of ROS through the activation of Nrf2 (Table 1) [36]. Moreover, HMOX1 was elevated and was responsible for ferroptosis in diabetic atherosclerosis [51]. Knockdown of HMOX1 mitigated ferroptosis and lipid peroxidation in diabetic human endothelial cells [51]. Collectively, iron metabolism and ferroptosis contribute to the pathogenesis of atherosclerosis, which are promising therapeutic targets that need further investigation.

### 3.2. Ferroptosis in Hypertension

Hypertension is a major risk factor for CVDs with increased peripheral vascular resistance as a result of the structural and functional changes in arteries [52]. Ferroptosis participates in the pathology of hypertension. It was evidenced in our prior work that iron levels were upregulated in the heart tissues of Ang II-infused hypertensive mice, accompanied by decreased levels of GPX4 and Nrf2 and increased malondialdehyde (MDA) (Figure 1) [12]. Treatment with elabela (ELA)-32 or Fer-1 improved cardiac function and mitigated myocardial hypertrophy and pathological remodeling through the inhibition of ferroptosis (Table 1) [12]. Recently, we have demonstrated that sirtuin 7 (SIRT7) or Fer-1 alleviated kidney impairments, fibrosis and renal ferroptosis in angiotensin II (Ang II)-mediated hypertensive mice (Table 1) [44]. Collectively, ferroptosis plays a key role in hypertensive myocardial remodeling and renal injury, indicating the potential therapeutic targets in the prevention and treatment of hypertension and hypertensive cardiorenal injury.

### 3.3. Iron Metabolism and Ferroptosis in Pulmonary Hypertension

Pulmonary arterial hypertension (PAH) is a vascular disease characterized by small pulmonary arteries remodeling, elevated pulmonary artery pressure and right ventricular hypertrophy [53,54]. PAH is a form of PH and is largely influenced by iron metabolism [55]. As reported, iron deficiency is involved in almost 40% of idiopathic PAH patients and is associated with reduced exercise capacity [56]. Improved quality of life and exercise capacity were associated with intravenous iron supplementation in patients with PAH, which may be the result of better oxygen transport within the skeletal muscle [55,57,58]. 

More evidence needs to be found on the role of iron deficiency or overload in the pathology of PH animal models [55]. Administration of deferoxamine (DFO) attenuated vascular remodeling in chronic hypoxia-induced PH rats [59]. However, pulmonary artery smooth muscle cell-specific iron deficiency was involved in the dysfunction of pulmonary vascular and the progression of PH in mice [60]. Ferroptosis participates in the occurrence and development of PH. Accumulated Fe^2+^ and reduced levels of GPX4 were found in monocrotaline-induced rats of PH (Figure 1) [13]. Fer-1 improved vascular remodeling and right ventricular function through the inhibition of ferroptosis, indicating the potential role of ferroptosis in PH (Table 1) [13]. Taken together, iron metabolism and ferroptosis are involved in the pathology of PH and more studies are needed to focus on the role of iron hemostasis in the development of PH.

### 3.4. Ferroptosis in Aortic Aneurysm and Dissection

Aortic aneurysm and dissection (AAD) are severe vascular diseases characterized by aortic medial degeneration [61,62]. Ferroptosis is critical in the occurrence and development of AAD. In Stanford type A aortic dissection patients, the levels of TfR and HMOX1 were upregulated and SLC7A11 and GPX4 were downregulated [46]. In addition, upregulated expression of HMOX1, TfR and 4-hydroxynonenal (4-HNE) was detected in β-Aminopropionitrile (BAPN)-induced AAD mice (Table 1) [46]. Administration of liproxstatin-1 (Lip-1) improved AAD incidence and death rates and alleviated medial degeneration through the suppression of ferroptosis (Table 1) [46]. Moreover, treatment with BRD4770 inhibited aortic dilation and reduced morbidity and mortality in BAPN-induced aortic dissection through the prevention of ferroptosis, lipid peroxidation and inflammation [63]. Therefore, targeting ferroptosis is a potential therapeutic strategy in the treatment of AAD.

### 3.5. Ferroptosis in Myocardial Ischemia/Reperfusion Injury

Myocardial infarction (MI) is the severe and irreversible injury of cardiomyocytes caused by sustained myocardial ischemia, which occurs due to the rupture or erosion of unstable coronary artery plaque [64]. Revascularization is an efficient strategy to rescue the ischemic myocardium from MI, while myocardial I/R injury inevitably aggravates myocardial impairments and reduces the therapeutic effects of reperfusion [40,64]. Iron metabolism-mediated ferroptosis participates in the occurrence of MI and myocardial I/R injury. The GPX4 levels were reduced in the left anterior descending ligation-induced MI mouse model, and depletion or inhibition of GPX4 led to the overproduction of lipid peroxide and ferroptosis-related H9c2 cell death [65]. In the myocardial I/R injury mice model, overexpression of GPX4 mitigated ferroptosis and myocardial impairments (Table 2) [15]. In addition, the upregulation of HMOX1 induced by hypoxia and hypoxia/reoxygenation(H/R) promoted heme degradation and iron accumulation in the endoplasmic reticulum and aggravated ferroptosis in cardiomyocytes [15]. Moreover, knockdown of TfR1 inhibited ferroptosis and blocked the elevated iron content and ROS production in H/R-treated H9c2 cells [66]. Overexpression of ubiquitin-specific protease 22 (USP22) suppressed ferroptosis-triggered cardiomyocyte death via the sirtuin 1/p53/SLC7A11 pathway [67]. In diabetic rats, activation of the Nrf2/FPN1 pathway mitigated myocardial I/R injury by preventing iron metabolism-mediated ferroptosis [68]. Taken together, iron metabolism-associated ferroptosis plays critical roles in the occurrence and development of MI and myocardial I/R injury, which could be therapeutic targets for the treatment of myocardial I/R injury-associated cardiac injury in the future.

### 3.6. Ferroptosis in Cardiomyopathy

#### 3.6.1. Sepsis-Induced Cardiomyopathy

Sepsis is caused by the dysregulation of inflammation due to an advanced immune reaction to infection [72]. Sepsis-induced cardiomyopathy (SIC) is a severe complication of sepsis with high mortality and poor prognosis characterized by the death of cardiomyocytes [16,17]. The hepcidin-FPN axis is significant in the regulation of iron homeostasis and is influenced by inflammation. In the state of sepsis, hepcidin is upregulated, which inhibits the release of iron, resulting in intracellular iron restriction [32]. Accumulated intracellular iron functions as a defense against external pathogens, while contributing to excessive ROS production and ferroptosis [72].

Ferroptosis is associated with the pathogenesis of SIC. Deposited myocardial iron, reduced GPX4 levels and upregulated prostaglandin endoperoxide synthase 2 (PTGS2) and FTH1 levels were found in LPS treated-SIC rats and could be blocked by Fer-1, suggesting the involvement of ferroptosis in SIC (Figure 1, Table 2) [16]. Accumulated iron and upregulated levels of PTGS2 and MDA were also found in LPS-induced mice and were rescued by dexrazoxane (DXZ) or Fer-1 (Figure 1) [17]. Mechanistically, LPS increased the expression of NCOA4 and intracellular Fe^2+^ levels but reduced ferritin levels [17]. NCOA4 degrades ferritin by ferritinophagy with the release of Fe^2+^, leading to excessive oxidative stress and ultimately ferroptosis [17]. Collectively, iron-mediated ferroptosis is critical in the development of SIC, suggesting a potential therapeutic target in the treatment of SIC-associated cardiac impairments.

#### 3.6.2. Doxorubicin-Induced Cardiomyopathy

DOX is a commonly used antitumor medicine with the side effect of dosage-dependent cardiotoxicity [70,71]. Ferroptosis was implicated in mice of DOX-induced cardiomyopathy characterized by elevated PTGS2 levels and the accumulation of lipid peroxidation which can be rescued by DXZ or Fer-1 [37]. Mechanistically, treatment with DOX led to cardiomyopathy through the accumulation of nonheme iron via heme degradation in response to Nrf2-associated upregulation of HMOX1, which can be rescued by HMOX1 antagonists or in Nrf2-deficient mice [37]. Mitochondria-dependent ferroptosis may play a key role in DOX-induced cardiac injury [42]. Administration of DOX in mice downregulated GPX4 levels and upregulated MDA levels [42]. Mitochondria-specific overexpression of GPX4 or downregulation of iron inhibited ferroptosis in cardiomyocytes, demonstrating the role of mitochondria in DOX-mediated ferroptosis (Table 2) [42]. Interestingly, FUN14 domain–containing 2 (FUNDC2) is a mitochondrial outer membrane protein, the knock-out of which alleviated DOX-induced cardiac impairments in mice and suppressed ferroptosis by regulating mitochondrial GSH (Table 2) [70]. MitoTEMPO, an antioxidant targeting mitochondria, has been implicated in rescuing DOX-induced cardiac injury [37]. Taken together, mitochondria-mediated ferroptosis is crucial in DIC and acts as a possible therapeutic target in the treatment of DOX-related myocardial injury.

### 3.7. Iron Metabolism and Ferroptosis in Heart Failure

Heart failure is characterized by significantly decreased cardiac output, the inability of myocardium and loss of cardiomyocyte cells [32,38,73]. Iron deficiency is commonly found in heart failure patients and is associated with worse outcomes [74,75,76]. Mechanistically, iron deficiency has been linked to impairments in oxidative metabolism, cellular energetics, and immune responses [76]. This leads to cardiac structural injury and dysfunction, reduced myoglobin oxygen storage, and decreased tissue oxidative capacity, all of which contribute to impaired mitochondria and left ventricular dysfunction [76]. Iron supplementation improved life quality and functional capacity in patients with heart failure and has been recommended in guidelines for the treatment of heart failure [32,74]. 

Ferroptosis is involved in the pathogenesis of heart failure. Activated ferroptosis was identified by downregulated GPX4 and FTH1 levels and upregulated 4-HNE levels in heart failure rat models induced by pressure overload (Figure 1) [18]. Knockdown of toll-like receptor 4 or NADPH oxidase 4 ameliorated left ventricular dysfunction and myocyte death by inhibiting autophagy and ferroptosis in heart failure (Table 2) [18]. Collectively, both iron metabolism and ferroptosis are implicated in the pathogenesis of cardiac dysfunction and injury, implying potential therapeutic targets for the prevention and treatment of heart failure.

## 4. Targeting Iron Metabolism and Ferroptosis in Cardiovascular Diseases

Dysregulated iron metabolism and ferroptosis participate in the physiological and pathological processes of CVDs. A variety of pharmacological compounds, including iron chelators and lipophilic antioxidants, possess the potential for the treatment of CVDs through the regulation of iron metabolism and ferroptosis (Table 3) [40]. 

Iron chelators have been demonstrated to mitigate myocardial iron overload and ameliorate cardiac dysfunction in thalassemia patients [24]. DFO is a clinically used iron chelator with a strong affinity for iron [69]. Intriguingly, DFO has been exhibited to alleviate ferroptosis and reduce myocardial infarct size in myocardial I/R injury by potentiating expression of GPX4 and preventing excessive lipid peroxidation (Table 2 and Table 3) [69]. In addition, DXZ is the only iron chelator approved by the Food and Drug Administration for the treatment of DIC in cancer patients [20]. Treatment with DXZ alleviated cardiac dysfunction through the inhibition of lipid peroxidation and ferroptosis in mice with DIC (Table 3) [37], and improved survival rates and myocardial function by preventing ferroptosis in LPS-induced SIC rats (Table 3) [17]. 

Fer-1 and Lip-1 are ferroptosis inhibitors and act as lipophilic antioxidants to suppress lipid peroxidation [19,20]. Fer-1 alleviated atherosclerosis in high-fat diet-induced ApoE^−/−^ mice through the suppression of ferroptosis by reducing iron content and inhibiting lipid peroxidation (Table 1 and Table 3) [9,20]. Furthermore, treatment with Fer-1 mitigated heart dysfunction in LPS-induced SIC rats by downregulating iron content, blocking decreased GPX4 levels, and alleviating inflammatory responses (Table 3) [16]. Additionally, Lip-1 improved cardiac dysfunction in myocardial I/R injury mice by reducing iron accumulation and mitigating ROS generation (Table 3) [77]. In BAPN-induced AAD mice, administration of Lip-1 downregulated the incidence and mortality of AAD and improved medial degeneration by blocking iron accumulation and excessive oxidative stress (Table 1 and Table 3) [46].

There are several other anti-ferroptosis molecules besides those mentioned above. In our published report, we demonstrated that administration of ELA-32 improved cardiac dysfunction and pathological myocardial remodeling by inhibiting ferroptosis in hypertensive mice (Table 1 and Table 3) [12]. In another study from our research group, ELA-32 treatment attenuated DOX-induced enhancement of oxidative stress and ferroptosis in primary rat aortic adventitial fibroblasts [80], indicating the potential role of ELA-32 in the treatment of ferroptosis-associated CVDs. Additionally, BRD4770 inhibited aortic dilation and decreased the mortality of AAD through the suppression of ferroptosis and oxidative stress (Table 3) [63]. Moreover, P22077, a inhibitor of ubiquitin-specific protease 7, mitigated myocardial I/R injury in rats by suppressing ferroptosis via the regulation of serum iron content and lipid peroxidation (Table 3) [66]. Furthermore, treatment with the NADPH oxidase 2 inhibitor Vas2870 alleviated cardiac injury in diabetic myocardial I/R injury by upregulating GPX4 levels and reducing ROS production (Table 3) [78]. Zinc protoporphyrin IX (ZnPP), an antagonist of HMOX1, has been demonstrated to block ferroptosis and alleviate DOX-induced heart impairments in DIC by suppressing HMOX1-mediated heme degradation (Table 3) [37]. Taken together, multiple compounds targeting iron metabolism and ferroptosis have the potential for the treatment of ferroptosis-mediated CVDs. However, the effectiveness still needs to be verified by more future studies.

## 5. Conclusions and Perspectives

Iron metabolism and homeostasis play critical roles in a variety of biochemical and physiological processes in the cardiovascular system. Ferroptosis is a newly discovered iron-related regulated cell death induced by iron accumulation and lipid peroxidation. Notably, dysregulation of iron metabolism and ferroptosis has been implicated in the pathogenesis of atherosclerosis, hypertension, pulmonary hypertension, heart failure, myocardial ischemia/reperfusion injury, aortic aneurysm and dissection and other CVDs. Furthermore, iron chelators and lipophilic antioxidants have been evidenced to suppress ferroptosis and lipid peroxidation in cardiomyopathy and other CVDs. Administration of the ferroptosis inhibitor Fer-1 mitigates cardiac structural injury and cardiovascular dysfunction in hypertension by blocking ferroptosis. Treatment with iron chelators and lipophilic antioxidants improves myocardial injury in cardiomyopathy by suppressing lipid peroxidation. More importantly, novel molecules such as ELA-32, SIRT7, Vas2870, BRD4770, P22077 and ZnPP can suppress ferroptosis and exert cardiovascular protective impacts on ferroptosis-related CVDs. Targeting iron metabolism and ferroptosis are potential therapeutic strategies in the prevention and treatment of hypertension and other CVDs.

## Figures and Tables

**Table 1:** Iron metabolism and ferroptosis in vascular diseases.

Experimental Model	Interventions	Effects and Mechanisms	Ref.
Mice with AS	Fer-1	Atherosclerotic lesion area↓, iron levels↓, GPX4↑, SLC7A11↑, MDA↓	[9]
ApoE^−/−^ mice	GPX4-Tg	Atherosclerotic lesions↓, lipid peroxidation↓	[41]
Ox-LDL-treated HCAECs	Overexpression of PDSS2	Cell death↓, iron content↓, GSH↑, Nrf2↑, ROS↓	[36]
Hypertensive mice	ELA-32	Cardiac hypertrophy and remodeling↓, myocardial fibrosis and dysfunction↓, SBP↓, iron levels↓, GPX4↑, Nrf2↑, MDA↓	[12]
Hypertensive mice	SIRT7	Kidney injury and dysfunction↓, renal fibrosis↓, GPX4↑, GSH/GSSG↑, Nrf2↑, NOX4↓, MDA↓	[44]
Rats with PH	Fer-1	Vascular remodeling↓, right ventricular function↑, iron content↓, GPX4↑, HMGB1↓, TLR4↓, NLRP3 inflammasome↓	[13]
Hypoxic PASMCs	SLC7A11 siRNA	GPX4↓, GSH/GSSG↓, MDA↑	[45]
Mice with AAD	-	Aortic diameter↑, HMOX1↑, TfR↑, lipid peroxidation↑	[46]
Mice with AAD	Lip-1	AAD incidence↓, medial degeneration↓, HMOX1↓, 4-HNE↓	[46]

Abbreviations: 4-HNE, 4-hydroxynonenal; AAD, aortic aneurysm and dissection; AS, atherosclerosis; ELA, elabela; Fer-1, ferrostatin-1; GPX4, glutathione peroxidase 4; GSH, glutathione; GSSG, glutathione disulfide; HCAECs, human coronary artery endothelial cells; HMGB1, high mobility group box-1 protein; HMOX1, heme oxygenase 1; Lip-1, liproxstatin-1; MDA, malondialdehyde; NLRP3, nucleotide-binding domain-like receptor protein 3; NOX4, nicotinamide adenine dinucleotide phosphate oxidase 4; Nrf2, nuclear factor erythroid 2-related factor 2; Ox-LDL, oxidized low-density lipoprotein; PASMCs, pulmonary artery smooth muscle cells; PDSS2, prenyldiphosphate synthase subunit 2; PH, pulmonary hypertension; Ref, references; ROS, reactive oxygen species; SBP, systolic blood pressure; SIRT7, sirtuin 7; SLC7A11, subunit solute carrier family 7 member 11; TfR, transferrin receptor; Tg, transgenic; TLR4, toll-like receptor 4; ↑upregulation, ↓downregulation.

**Table 2:** Iron metabolism and ferroptosis in heart diseases.

Experimental Model	Interventions	Effects and Mechanisms	Ref.
Rats			
MIRI	-	Infarct area↑, CK activity↑, iron content↑, GPX4↓, ACSL4↑, ROS↑	[69]
MIRI	DFO	Infarct size↓, iron levels↓, GPX4↑, lipid peroxidation↓	[69]
SIC	-	Myocardial function↓, iron levels↑, GPX4↓, PTGS2↑	[16]
HF	TLR4-siRNA/NOX4-siRNA	Heart function↑, myocyte death↓, intracellular iron↓, GPX4↑, 4-HNE↓	[18]
Mice			
MIRI	ELAVL1-siRNA	Infarct size↓, iron levels↓, GPX4↑, ROS↓	[14]
MIRI	GPX4-Tg	Myocardial impairments↓, lipid peroxides↓, TUNEL+ cells↓	[15]
DIC	GPX4-Tg	Heart impairments↓, LVEF↑, MDA↓	[42]
DIC	FUNDC2-KO	Cardiac function↑, cardiac fibrosis↓, 4-HNE↓, PTGS2↓	[70]
DIC	rAAV9-PRMT4	Myocardial injury↑, cardiac function↓, GPX4↓, GSH↓, ROS↑	[71]

Abbreviations: 4-HNE, 4-hydroxynonenal; ACSL4, acyl-coenzyme A synthetase long-chain family member 4; CK, creatine kinase; DFO, deferoxamine; DIC, DOX-induced cardiomyopathy; ELAVL1, embryonic lethal-abnormal vision like protein 1; FUNDC2, FUN14 domain–containing 2; GPX4, glutathione peroxidase 4; GSH, glutathione; HF, heart failure; KO, knock-out; LVEF, left ventricular ejection fraction; MDA, malondialdehyde; MIRI, myocardial ischemia/reperfusion injury; NOX4, nicotinamide adenine dinucleotide phosphate oxidase 4; PRMT4, protein arginine methyltransferase 4; PTGS2, prostaglandin endoperoxide synthase 2; rAAV9, recombinant adeno-associated virus type 9; Ref, references; ROS, reactive oxygen species; SIC, sepsis-induced cardiomyopathy; Tg, transgenic; TLR4, toll-like receptor 4; ↑upregulation, ↓downregulation.

**Table 3:** Targeting for iron metabolism and ferroptosis in CVDs.

Dis.	Interventions	Targets	Effects and Mechanisms	Ref.
AS	Fer-1	Inhibit lipid peroxidation	Atherosclerotic lesion↓, iron accumulation↓, GSH↑, SCL7A11↑, lipid peroxidation↓	[9]
HT	ELA-32	Inhibit ferroptosis	Myocardial fibrosis and dysfunction↓, SBP↓, iron levels↓, ROS↓, GPX4↑, Nrf2↑	[12]
HT	Fer-1	Inhibit lipid peroxidation	Cardiac hypertrophy and remodeling↓, GPX4↑, MDA↓	[12]
PH	Fer-1	Inhibit lipid peroxidation	Right ventricular hypertrophy↓, iron levels↓, HMGB1↓, TLR4↓, NLRP3 inflammasome↓	[13]
AAD	Lip-1	Inhibit lipid peroxidation	AAD incidence↓, mortality↓, TfR↓, HMOX1↓, lipid peroxidation↓	[46]
AAD	BRD4770	Inhibit ferroptosis	AAD mortality↓, aorta dilation↓, medial degradation↓, HMOX1↓, SLC7A11↑, FSP1↑, lipid peroxidation↓, neutrophil infiltration↓	[63]
MIRI	P22077	Inhibit USP7	Infarct size↓, cardiac fiber loss↓, iron content↓, TfR1↓, GPX activity↑, ACSL4↓, lipid peroxidation↓	[66]
MIRI	DFO	Iron chelation	Infarct size↓, CK activity↓, iron content↓, GPX4↑, ACSL4↓, lipid peroxidation↓	[69]
MIRI	Lip-1	Inhibit lipid peroxidation	Myocardial infarct size↓, mitochondrial structural integrity↑, GPX4↑, ROS↓, VDAC1↓	[77]
DIMI	Vas2870	Inhibit NOX2	Cardiac injury↓, GPX4↑, oxidative stress↓,	[78]
SIC	Fer-1	Inhibit lipid peroxidation	Cardiac function↑, iron content↓, GPX4↑, PTGS2↓, inflammatory cell infiltration↓, TLR4↓, NF-κB↓	[16]
SIC	DXZ/Fer-1	Iron chelation/Inhibit lipid peroxidation	Survival rate↑, cardiac injury↓, ferric iron↓, PTGS2↓, MDA↓, inflammatory cells↓	[17]
DIC	ZnPP	Inhibit HMOX1	Cardiac injury↓, MDA↓, 4-HNE↓, PTGS2↓	[37]
DIC	DXZ/Fer-1	Iron chelation/Inhibit lipid peroxidation	Myocardial hypertrophy↓, cardiac function↑, lipid peroxidation↓, PTGS2↓	[79]

Abbreviations: 4-HNE, 4-hydroxynonenal; AAD, aortic aneurysm and dissection; ACSL4, acyl-coenzyme A synthetase long-chain family member 4; AS, atherosclerosis; CK, creatine kinase; CVDs, cardiovascular diseases; DFO, deferoxamine; DIC, DOX-induced cardiomyopathy; DIMI, diabetes-induced myocardial infarction; DIS, disease; DXZ, dexrazoxane; ELA, elabela; Fer-1, ferrostatin-1; FSP1, ferroptosis suppressor protein 1; GPX, glutathione peroxidase; GPX4, glutathione peroxidase 4; GSH, glutathione; HMGB1, high mobility group box-1 protein; HMOX1, heme oxygenase 1; HT, hypertension; Lip-1, liproxstatin-1; MDA, malondialdehyde; MIRI, myocardial ischemia/reperfusion injury; NF-κB, nuclear factor kappa-B; NLRP3, nucleotide-binding domain-like receptor protein 3; NOX2, nicotinamide adenine dinucleotide phosphate oxidase 2; Nrf2, nuclear factor erythroid 2-related factor 2; PH, pulmonary hypertension; PTGS2, prostaglandin endoperoxide synthase 2; Ref, references; ROS, reactive oxygen species; SBP, systolic blood pressure; SIC, sepsis-induced cardiomyopathy; SLC7A11, subunit solute carrier family 7 member 11; TfR, transferrin receptor; TLR4, toll-like receptor 4; USP7, ubiquitin-specific protease 7; VDAC1, voltage-dependent anion channel 1; ZnPP, zinc protoporphyrin IX; ↑upregulation, ↓downregulation.

## Data Availability

Not applicable.
